# The consequences of the extremely low frequency electromagnetic field (ELF-EMF) exposure on physiological processes in the uterus: novel insights and implications

**DOI:** 10.3389/fphys.2026.1753084

**Published:** 2026-03-11

**Authors:** Mohsen Khodadadi, Agata Zmijewska, Anita Franczak

**Affiliations:** Department of Animal Anatomy and Physiology, Faculty of Biology and Biotechnology, University of Warmia and Mazury in Olsztyn, Olsztyn, Poland

**Keywords:** apoptosis, cell proliferation, endometrium, epigenetic modifications, extremely low frequency electromagnetic fields (ELF-EMFs), myometrium, steroidogenesis, transcriptomic profile

## Abstract

Extremely low frequency electromagnetic field (ELF-EMF), ranging from 1 Hz to 300 Hz, is prevalent in modern environments, yet its biological effects on the production remain insufficiently explored. This mini-review summarizes recent findings on ELF-EMF-induced alterations in the biological processes involved the uterus. It has been documented that the exposure to the ELF-EMF has been linked to significant changes in alterations of transcriptomic profile, histone modifications, DNA methylation, and microRNA pathways in the uterus, which may disrupt uterine contractility, secretory activity and hormonal signalling. Furthermore, the ELF-EMF influences myometrial and endometrial steroidogenesis, interferes with calcium ion channel regulation, elevates oxidative stress, apoptosis and cell proliferation, raising concerns about uterine tissues integrity. While direct evidence of ELF-EMF-induced tumorigenesis in the myometrium and the endometrium is lacking, its potential role in disrupting the mRNA transcript abundance involved in oxidative stress, apoptosis and cell proliferation underscores the need for further investigation. This review highlights the potential reproductive risks associated with the ELF-EMF exposure and calls for additional *in vivo* studies to elucidate its long-term effects on female fertility and reproductive health.

## 1 Introduction

Electromagnetic fields (EMFs) have become a significant environmental concern in recent decades due to their omnipresence in modern life (Singh and Kapoor, 2014). Among the types of the EMFs, extremely low frequency electromagnetic field (ELF-EMF), defined as fields with frequencies between 1 Hz and 300 Hz, has gained particular interest for its potential biological effects on living organisms (Kheifets et al., 2006; Schuermann and Mevissen, 2021). The presence of the ELF-EMF in everyday environments, ranging from power lines to household appliances, means that long term exposure is almost inevitable (Ghanb et al., 2022; Jafari et al., 2024).

**FIGURE 1:**
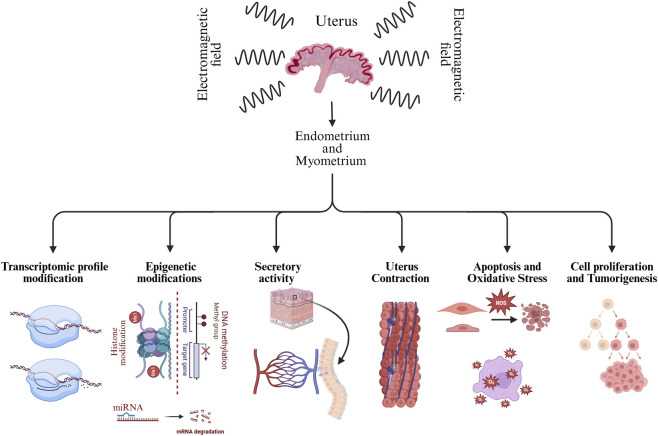
Representing the effects of extremely low-frequency electromagnetic field (ELF-EMF) exposure on uterine tissues (endometrium and myometrium). Molecular and cellular mechanisms shown (including transcriptomic, epigenetic, secretory, apoptotic, oxidative stress, and proliferative responses) are supported by experimental evidence from in vitro and/or *in vivo* studies. The effect on uterine contractility is shown as a hypothesized outcome, derived from related findings rather than directly assessed.

While the effects of the ELF-EMF on the nervous system have been well documented (Lai, 2022; Eskandani and Zibaii, 2023; Klimek and Rogalska, 2021), its influence on reproduction, and specifically the activity of the myometrium and the endometrium, has only recently been explored in depth (Wydorski et al., 2024a; Franczak et al., 2023; Kozlowska et al., 2022; Drzewiecka et al., 2021a; Kozlowska et al., 2021a; Kozlowska et al., 2021b; Wydorski et al., 2023; Franczak et al., 2020). Given the importance of the myometrium and the endometrium in the female fertility and pregnancy maintenance, understanding how the ELF-EMF exposure might disrupt their function is critical. The myometrium, the outer smooth muscle layer of the uterus, is essential for female reproductive health as it regulates uterine contraction, supports structural integrity, produces prostaglandins (PGs) and steroid hormones, and facilitates key processes involved in female reproductive system such as regulation of the estrous cycle, sperm transport, embryo implantation, maintenance of pregnancy, and labor (Young, 2007; Wray and Prendergast, 2019; Shynlova et al., 2020; Franczak and Kotwica, 2010; Franczak et al., 2006). The endometrium, the inner epithelial lining of the uterus, is another crucial tissue of the uterus, as it undergoes cyclical changes and remodeling in the response to hormonal cues, produces PGs and steroid hormones, regulates implantation, and supports corpus luteum maintenance during early pregnancy or its regression during luteolysis (Strowitzki et al., 2006; Deligdisch, 2000; Poyser, 1995; Franczak, 2008; Ashworth et al., 2006; Ziecik et al., 2016). Together, the myometrium and the endometrium play crucial roles in regulating uterine function throughout the reproductive cycle, pregnancy maintenance and labor. This review aims to summarize and assess the existing literature on the consequences of ELF-EMF radiation in both the myometrium and the endometrium, focusing on key biological processes such as transcriptomic activity, epigenetic modifications, secretory activity, uterus contractility, apoptosis, oxidative stress, and cell proliferation (Figure 1), which are mostly focusing on animal models due to ethical and practical constraints of directly studying these effects in humans.

## 2 Transcriptomic profile modification in the endometrium and the myometrium induced by the ELM-EMF

Transcriptomic profile modification refers to changes involved in the type and level of RNA transcriptions. Various biological processes and external stimuli can result in these modification, and by modifying the transcriptomic profile they play a crucial role in regulating gene expression and consequently cellular function (Kumar et al., 2016).

It has been reported that the *in vitro* treatment of ELF-EMF (50 Hz, 2 h) can lead to significant changes in the transcriptomic profile in pig’s endometrium. It has been determined alterations in the expression of 1561 transcriptionally active regions (TARs), with 461 differentially expressed genes (DEGs), with 34% rate of being upregulated and 66% downregulated in the tissue (Kozlowska et al., 2021b). These changes are associated with biological processes such as cell proliferation and metabolism, which are critical for proper functionality of the endometrium. It has also been documented that the ELF-EMF exposure can alter the expression of various genes and non-coding RNAs in the endometrium, including caspase 3 (*CASP3)*, caspase 7 (*CASP7)*, cell death–inducing DFFA-like effector b (*CIDEB)*, growth arrest and DNA-damage-inducible gamma (*GADD45G)*, enhancer of zeste homolog 2 (*EZH2)*, ubiquitin-like with PHD and ring finger domains 1 (*UHRF1)*, and methyl-CpG binding domain protein 1 (*MBD1)*, connected with oxidative stress responses and epigenetic modification, which can lead to disruption of endometrial normal physiological functions (Wydorski et al., 2024a; Wydorski et al., 2024b). Furthermore, it has been observed that ELF-EMF exposure can affect the expression of non-coding RNAs, including long non-coding RNAs (lncRNAs) and microRNAs (miRNAs) biogenesis (Kozlowska et al., 2021b; Wydorski et al., 2024b), which are essential for gene regulation (Patil et al., 2014). These observed transcriptomic changes can have significant consequences for reproductive health. For example, dysregulated expression of endometrial genes involved in cell proliferation Tumor Protein 53 (*TP53*), Cyclin E2, B-cell lymphoma 2 (*BCL2*) and metabolism (Acyl-CoA Synthetase Long Chain Family Member 4 (*ACSL4*), Nicotinamide N-Methyltransferase (*NNMT*), Solute Carrier Family 26 Member 2 (*SLC26A2*)) during the peri-implantation period could result in impaired tissue receptivity, which potentially affects implantation and pregnancy outcomes (Ye and Dimitriadis, 2025; Wang et al., 2024; Matsuyama et al., 2024). Moreover, by combining different transcriptomic studies (Kozlowska et al., 2021b; Franczak et al., 2025) it can be said that epigenetic changes caused by the ELF-EMF radiation might be a potential contributor to the development of reproductive disorders.

While fewer studies have focused specifically on the myometrium, the current evidence suggests that ELF-EMF exposure can also induce transcriptomic alteration in this tissue (Drzewiecka et al., 2021a). For example, it has been documented that the ELF-EMF (50 Hz, 2 h) *in vitro* treatment could affect the expression of 215 transcript active regions (TARs), and among these regions, 90 genes involved in protein coding were differentially expressed, categorized mostly to gene ontology terms connected with defense and immune responses, apoptosis, and inflammation, i.e. homeobox D13 (*HOXD13)*, prodynorphin (*PDYN)*, vascular cell adhesion molecule 1 (*VCAM1)*, interleukin 15 (*IL15)*, signal transducer and activator of transcription 5A (*STAT5A)*, tumor necrosis factor (*TNF)*, and early growth response 2 (*EGR2)* (Drzewiecka et al., 2021a).

It was found that the ELF-EMF exposure can disrupt the function of voltage-gated ion channels, which leads to changes in intracellular ionic concentrations (Panagopoulos et al., 2025). This disruption can result in the overproduction of reactive oxygen species (ROS), which can damage DNA and disrupt normal cellular function (Panagopoulos et al., 2021). The resulting oxidative stress can trigger signaling pathways that alter gene expression, contributing to the transcriptomic changes (Li J. et al., 2018; Siauciunaite et al., 2019). Furthermore, it has been observed that after the exposure to ELF-EMF the expression of genes encoding serpin family E member 1 (SERPINE1) and plasminogen activator (PLAT), involved in fibrinolysis and cell adhesion, were changed in both conceptuses and the endometrium, which can indicate the potential adverse effect of ELF-EMF on disrupting embryo–maternal communication (Franczak et al., 2025).

## 3 Epigenetic modifications induced by ELF-EMF in the endometrium and the myometrium

Epigenetic modifications refer to the alterations in gene regulation and expression without altering the DNA sequence through processes such as histone acetylation and methylation, micro RNA biogenesis, and DNA methylation, which regulate gene transcription and playing a critical role in cellular function and response to environmental stimuli (Vinci, 2012). The ELF-EMF exposure has been associated with changes in the epigenetic landscape both in the endometrium and the myometrium, potentially affecting reproductive outcomes (Franczak et al., 2023).

DNA methylation is an epigenetic modification, where a methyl group (–CH_3_) is added to the DNA molecule, critical for gene silencing and cellular differentiation (Mattei et al., 2022). It has been shown that epigenetic changes, specifically DNA methylation, are closely associated with aging and numerous disease (Mc Auley, 2021; Wang et al., 2022; Smith et al., 2024). Furthermore, a number of studies indicated that the ELF-EMF can induce its abnormal effects through targeting DNA methylation level (Colciago et al., 2024; Sendera et al., 2024). The ELF-EMF exposure has been shown to modulate epigenetic mechanisms, including DNA methylation and histone modification (Wydorski et al., 2024b; Manser et al., 2017). These changes can influence the expression level of genes (such as Cyclin D2, DNA-methyltransferase 1 (*DNMT1*), microRNA-21 (*miR-21*), Leukemia Inhibitory Factor (*LIF*), Progesterone Receptor (*PGR*) and etc.) involved in cell proliferation, differentiation, survival, and potentially lead to long-term effects on uterine function (Shayeghan et al., 2021; Giorgi and Del Re, 2021). It has been shown that the ELF-EMF exposure during the peri-implantation period may alter the epigenetic regulation by affecting the expression of DNA (cytosine-5)-methyltransferases (DNMTs), implying a potential influence on DNA methylation patterns in the endometrium and the myometrium (Franczak et al., 2023; Wydorski et al., 2024b). Interestingly, it has been reported that in the endometrium exposed to the ELF-EMF increased methylation of *EGR2,* inhibitor of DNA binding 2 (*ID2)*, helix-loop-helix *(HLH)* protein, and prostaglandin E receptor 4 (*PTGER4)* and decreased methylation of interleukin 1 receptor accessory protein (*IL1RAP)* and nitric oxide synthase 3 (*NOS3)* were observed (Wydorski et al., 2023). These genes with altered methylation level encode proteins connected with important biological processes such as tissue secretory activity, generation of free radicals, cell proliferation and differentiation, and signal transduction (Kozlowska et al., 2021b; Zhu et al., 2007; Yue et al., 2018; Porasuphatana et al., 2003). Observed changes in the level of DNA methylation appear to be dangerous for proper endometrial activity and receptivity during the peri-implantation period (Wydorski et al., 2023). These modifications may alter the transcription of genes critical for maintaining uterine secretory activity and responding to hormonal cues (Plunk and Richards, 2020; Ret et al., 2021), and also they can affect the implantation (Kong et al., 2019; Liu et al., 2020). Furthermore, it has been observed that in the myometrium exposure to the ELF-EMF modifies the methylation status of promoter regions in several genes, including *STAT5A*, *EGR2*, *PDYN*, *IL15*, *TNF*, and ubiquitin associated protein 2 (*UABP2*), while transcriptional changes in Hyaluronan and Proteoglycan Link Protein 1 (*HAPLN1*) appear to occur independently of promoter methylation. These genes are mostly involved in various biological processes such as cell proliferation and differentiation (in general cell growth), nervous system signalling, apoptosis and immune regulation in cells (Franczak et al., 2023). Moreover, histone modification commonly coincides with DNA methylation and contributes to alterations in gene transcription patterns therefore, changes in histone modification could also result in dysregulation of genes responsible for maintaining uterine relaxation during pregnancy (Liu et al., 2020; Kondo, 2009). Therefore, these findings suggest that during the peri-implantation period the ELF-EMF can lead to disturbances in the epigenetic mechanisms in the uterus and furthermore implantation.

There have been several studies indicating that the ELF-EMF can affect microRNA pathways and its biogenesis (Li H. et al., 2018; Liu et al., 2015; Heidari et al., 2021). MicroRNAs (miRNAs) are small noncoding RNAs that regulate gene expression at the posttranscriptional level (Lu and Rothenberg, 2018). The biogenesis of miRNAs is regulated by key processing enzymes dicer 1 *(DICER1)* and DiGeorge syndrome critical region 8 *(DGCR8)* (Condello et al., 2024). Several studies have suggested that the ELF-EMF exposure affects miRNA biogenesis pathways by altering the expression of miRNA processing enzymes, which can disrupt the regulation of genes involved in cell proliferation, apoptosis, and hormone signalling in the endometrium and the myometrium (Wydorski et al., 2024b).

In summary, the ELF-EMF may induce epigenetic changes and potentially disturb the transcriptomic profile of the uterus, impairing cell proliferation, angiogenesis, and tissue receptivity by disrupting the hormonal environment necessary for successful implantation and maintenance of pregnancy, which potentially may influence pregnancy outcomes. Therefore, the exposure of the endometrium and myometrium to the ELF-EMF may lead to epigenetic changes that could disrupt the transcriptomic profile of the uterus. This disruption may impair genes involved in cell proliferation, angiogenesis, and endometrial tissue receptivity by altering the hormonal environment essential for successful implantation and pregnancy maintenance.

## 4 Uterine steroidogenesis and ELF-EMF-induced consequences

The proper steroidogenesis in the myometrium and the endometrium is essential to maintaining uterine function, particularly during the early stages of pregnancy (Shynlova et al., 2009; Franczak and Kotwica, 2010). Myometrial and endometrial cells secrete a variety of hormones such as PGs, growth factors, cytokines and steroid hormones that affect vascular development, cell proliferation, adhesion, and migration, which are crucial for implantation and maintaining the estrous or menstrual cycle and regulate the proper intra-uterine environment (Shynlova et al., 2009; Billhaq et al., 2020; Dorniak and Spencer, 2018; Sugimoto et al., 2015).

Steroid hormones are key regulators of uterine function, which work in coordination to prepare the endometrium and the myometrium for the implantation and to support early pregnancy (Noyola-Martínez et al., 2019; Solano and Arck, 2020). Progesterone plays a crucial role in transforming the endometrium into a secretory state, while stabilizing the tissue, and suppressing uterine contractions, all of which are necessary for successful implantation of embryo and the maintenance of pregnancy (Ozturk and Demir, 2010). Estrogen promotes the proliferation and thickening of the endometrial tissue during the follicular phase, increases blood flow to uterus, and upregulates progesterone receptors, by doing so, it will prepare the uterus for the actions of progesterone (Ozturk and Demir, 2010). Moreover, androgens play various important roles in pregnancy by influencing placental development, maternal vascular function, and the process of parturition (Parsons and Bouma, 2021; Kumar et al., 2024; Makieva et al., 2014). These hormones, which sometimes overshadowed by estrogens and progesterone, are crucial for maintaining pregnancy and ensuring successful embryo development.

The effect of the ELF-EMF exposure on steroidogenic and secretory activity of the uterus was studied mainly in animal models especially pigs, and still needs to be investigated with more depth for better understanding (Kozlowska et al., 2021a; Franczak et al., 2020; Jangid et al., 2023; Franczak et al., 2022; Koziorowska et al., 2018).

It was found that exposure of uterine tissues to the ELF-EMF interferes with hormonal signalling, particularly affecting the synthesis and secretion of steroid hormones, which can result in disturbance in the proper function of the uterus, especially during estrous cycle and early pregnancy (Kozlowska et al., 2022; Kozlowska et al., 2021a; Franczak et al., 2020; Jangid et al., 2023; Franczak et al., 2022; Koziorowska et al., 2018). It has been documented that the ELF-EMF exposure alters estrogens release in the endometrium and androgen release in the myometrium, with potential involvement for uterine receptivity and early pregnancy maintenance (Franczak et al., 2020; Koziorowska et al., 2018). Therefore, any disruption in uterine steroids production or signalling may potentially affect the fertility and the ability to sustain pregnancy (Solano and Arck, 2020; Da Silva et al., 2024; DeMayo, 2024).

Furthermore, it has been shown that the ELF-EMF at the frequency of 50 Hz and 120 Hz alters the expression of *CYP19A3* and *HSD17B4*, which are key genes responsible for estrogen synthesis and alteration, accompanied by changes in aromatase and 17β-HSD protein levels and modulated release of estrone (E_1_) and estradiol (E2) in porcine endometrium and myometrium during the peri-implantation period (Koziorowska et al., 2018; Drzewiecka et al., 2021b). It has been also reported that ELF-EMF exposure can affect and disrupt the secretion of estradiol-17β (E_2_) in the uterine tissues (Koziorowska et al., 2018). Alterations in E_2_ secretion could compromise the uterine environment, thereby reducing the likelihood of successful implantation and increasing the risk of pregnancy complications, highlighting significant concerns for fertility and overall reproductive health (Parisi et al., 2023; Boucher et al., 2022). It has been also shown that exposure to ELF-EMF at the frequency of 50 Hz for 2–4 h can lead to excessive production of oestrogen which can result in serious pregnancy complications in roe deer (Koziorowska et al., 2024). While several studies in pigs and other animal models have shown that ELF-EMF exposure disrupts steroidogenic pathways by altering key enzymes and modulating estrogen and androgen secretion, findings in rodent models are less consistent. For instance, Aydın et al. (2009) reported that prolonged exposure of female rats to 50 Hz ELF-EMF did not significantly alter serum P_4_ and E_2_ levels, nor did it cause marked histopathological changes in the uterus or ovaries (Aydin et al., 2009). However, they observed a significant reduction in plasma catalase activity, indicating increased oxidative stress. These results suggest that ELF-EMF may compromise uterine function through oxidative imbalance, which in turn could impair uterine receptivity and early pregnancy maintenance (Aydin et al., 2009; Gye and Park, 2012).

## 5 Uterus contraction and the impact ELF-EMF radiation

Uterine contractions are fundamental to reproductive processes such as menstruation, embryo transport from the Fallopian tube, maintaining early pregnancy and labor, which are primarily generated by the myometrial layer of uterus (Young, 2007; Shynlova et al., 2020; McEvoy and Sabir, 2018). The regulation of myometrial contraction is complex and involves ion channels, receptors as well as hormonal, neural, and local cellular signalling pathways (Shynlova et al., 2009; Pehlivanoğlu et al., 2013; Rosen and Yogev, 2023; Wray et al., 2001). It has been found that the ELF-EMF exposure may interfere with these pathways, which can be mechanistically and inferentially linked to potential complications during pregnancy or labor (Bertagna et al., 2021).

Moreover, it has been documented that exposure to ELF-EMF has been linked to alterations in the expression of key steroid and gonadotropin hormones (Jangid et al., 2023) which can possibly affect the regulation of myometrial contractility. Such hormonal changes can disrupt contraction patterns, with animal studies indicating reduced delivery rates and a higher incidence of preterm births following ELF-EMF exposure (Zhang et al., 2016; Zangeneh and Hantoushzadeh, 2023). Ion channel activity, particularly those involved in calcium signalling, is critical for myometrial contraction and proper performance (Wray and Arrowsmith, 2021). Furthermore, it also has been shown that the ELF-EMF can have a potential adverse effect dysfunction of voltage-gated ion channels (VGICs) in cell membranes through the ion forced oscillation mechanism, which can affect various ions regulation, such as calcium, sodium, and potassium (Panagopoulos et al., 2022). Disruptions in ion channel function can lead to altered uterine contractility, potentially complicating labor or leading to preterm contractions (Zangeneh and Hantoushzadeh, 2023; Wray and Arrowsmith, 2021). Thereby, we believe that these changes can potentially alter uterine contraction dynamics. Also, it has been reported that the ELF-EMF can alter the calcium signaling (Liburdy, 1992), which may affect smooth muscle contractility and the secretory activity (Hill-Eubanks et al., 2011; Kuo and Ehrlich, 2015; Arnold et al., 2021). Notably, it has been documented that PGs possess an important role in modulating myometrial contractility (Chiossi et al., 2012), but until now there are no specified documents indicating the consequences of the ELF-EMF radiation on PGs synthesis in the myometrium.

The regulation of uterine contraction is also influenced by β-adrenergic receptors (Drozd and Moroz, 2020; Modzelewska et al., 2021). The role of β-adrenergic receptors in relaxing the myometrium during pregnancy, preventing premature contractions, has been previously documented (Asif et al., 2023). It has been documented that the ELF-EMF exposure may contribute to modulating these receptors or the associated signalling pathways in the heart alongside other environmental stimuli (Cornacchione et al., 2016). Thereby, we suspect that there might be a potential occurrence of the same pattern in myometrium and increasing risk of preterm labor or dysfunctional uterine contractions.

Moreover, it has been also proposed that ELF-EMF treatment may interfere with transient receptor potential (TRP) channels expression or function in the endometrium (Wydorski et al., 2024a). Members of the TRP channel subfamily play an important role in regulating calcium homeostasis in myometrial cells by intervening in store-operated calcium entry (SOCE) and receptor-operated calcium entry (ROCE) pathways (Ku et al., 2006; Tsagareli et al., 2020). Disruption of TRP channels can impair calcium entry, leading to altered intracellular calcium fluctuations and disturbed myometrial contractility (Wray and Prendergast, 2019). Despite the lack of information about disruption of calcium homeostasis in myometrial cells due to the effects of the ELF-EMF on TRP channels, by referring to other studies which have been conducted on this matter (Wydorski et al., 2024a; Ma et al., 2016), there might be a possibility that ELF-EMF can lead to abnormal myometrial function and contractility through this process that can have a direct effect on pregnancy out comes, however for precise conclusion needs further investigations. To summarize, disruptions in ion channel function and hormonal signalling pathways due to the ELF-EMF radiation may lead to altered uterine contractility, having serious implications for pregnancy maintenance, potentially complicating labor, increasing the risk of preterm uterine contractions which results in pregnancy lost.

## 6 The effect of the ELF-EMF on apoptosis and oxidative stress in endometrial and myometrial cells

Apoptosis, or programmed cell death, and oxidative stress are critical for maintaining tissue homeostasis. Excessive apoptosis or oxidative stress in the myometrium and the endometrium can lead to tissue dysfunction and impaired reproductive outcomes (Wydorski et al., 2024a; Agarwal et al., 2005; Lu et al., 2018). BCL2 associated X *(BAX)* and B-cell CLL/lymphoma 2 *(Bcl-2)* are two key regulatory proteins in the intrinsic apoptotic pathway, with opposing roles in controlling cell death by promoting and inhibiting it (Palabiyik, 2025; Singh et al., 2019). BAX promotes apoptosis by permeabilizing the mitochondrial outer membrane, enabling the release of cytochrome “c” and caspase activation, while Bcl-2 inhibits this process by preventing BAX activation and maintaining mitochondrial integrity (Seervi et al., 2011). Dysregulation in the balance between these proteins can determine a cell’s fate and result in various diseases (Qian et al., 2022).

It has been demonstrated that the ELF-EMF treatment alters apoptotic gene expression in the endometrium, notably downregulating *CASP7* and upregulating *CIDEB*, which may affect cell detachment and tissue remodeling (Wydorski et al., 2024a). Similar mechanisms could also be at play in the myometrium, where apoptosis and cell proliferation are crucial for tissue remodelling, particularly during the estrous cycle or pregnancy, but there is a need for further investigation about it (Garip and Akan, 2010). It was reported that ELF-EMF also upregulates *CASP3* and caspase 9 (*CASP9)*, genes that encode important mediators of apoptosis, further exacerbating cell death in the endometrium (Wydorski et al., 2024a). Furthermore, it has been documented that ELF-EMF exposure can result in changes in the regulation of some proapoptotic markers, such as BAX and some antiapoptotic markers like BCL2 in the endometrium (Jangid et al., 2023; Barati et al., 2021). Also, it has been suggested that ELF-EMF may increase apoptosis in myometrial cells, which leads to excessive cell death (Wydorski et al., 2024a; Barati et al., 2021; Korshunova et al., 2021), potentially compromising tissue integrity and uterine function during pregnancy (Xie et al., 2024). However, the consequences of ELF-EMF regarding these mechanisms in the myometrium are still unknown and they need to be further investigated (Kozlowska et al., 2021b; Asghari et al., 2016).

The oxidative stress in any tissue results from an imbalance between reactive oxygen species (ROS) production and antioxidant defences (Ozougwu, 2016). The natural production of ROS is entangled with various developmental processes during estrus cycle and pregnancy, including oocyte maturation, luteolysis, and embryo implantation (Hussain et al., 2021). However, excessive ROS production can interfere with these processes, leading to reproductive failure (Hussain et al., 2021). The ELF-EMF exposure has been linked to increased ROS production in myometrial cells, that might lead to cellular damage, mitochondrial dysfunction (Santini et al., 2018; Marín et al., 2020), and ultimately contribute to conditions like preeclampsia (Mandò et al., 2014) and intrauterine growth restriction (IUGR) during pregnancy (Tian et al., 2023). Moreover, it has been shown that heightened oxidative stress can disrupt normal cellular functions and may contribute to inflammation and uterine tissue damage (Burton and Jauniaux, 2011; Song et al., 2022). The involvement of NOS3 in ELF-EMF-induced oxidative stress further highlights the potential for ELF-EMF to disrupt redox homeostasis in the endometrium (Wydorski et al., 2024a). These observations are supported by a study reporting that hypomethylation of the NOS3 promoter induced by the ELF-EMF is associated with its increased expression in the endometrium (Wydorski et al., 2024a), which can potentially lead to excessive nitric oxide production and nitroxidative stress and lead to inflammatory conditions (Wydorski et al., 2023). Moreover, it has been suggested that the ELF-EMF exposure may disrupt mitochondrial function, leading to excessive ROS accumulation and cellular injury in the endometrium (Wydorski et al., 2024a; Barati et al., 2021). Despite the upregulation of antioxidant enzymes like SOD2 and GPX, these compensatory mechanisms fail to mitigate the oxidative damage caused by ELF-EMF (Barati et al., 2021; Georgiou, 2010). In line with these observations, it has been reported that EMF exposure triggers inflammatory responses in uterine tissues, which are linked to excessive oxidative stress and cell apoptosis (Alchalabi et al., 2016). Therefore, by amplifying apoptotic pathways and overwhelming antioxidant defences, the ELF-EMF could severely impair the balance necessary for normal uterine function during pregnancy (Wydorski et al., 2024a; Alchalabi et al., 2016; Toboła-Wróbel et al., 2020).

## 7 Uterine cell proliferation and tumorigenesis in the presence of the ELF-EMF

The proper cell proliferation is necessary for tissue repair and regeneration, but unregulated proliferation can lead to pathological conditions such as fibroids or malignancy (Reis et al., 2016; Sobolewski et al., 2010). The ELF-EMF exposure has been shown to influence cell proliferation in the myometrium and the endometrium (Franczak et al., 2023; Wydorski et al., 2024b). Also, it has been demonstrated that epigenetic changes associated with ELF-EMF affecting microRNA, DNA methylation and histone modifications could affect the proliferation and the homeostasis of cancer cells (Shayeghan et al., 2021). Importantly, as discussed previously, it has been documented that ELF-EMF can modulate the transcription of steroidogenic genes and disrupt steroids synthesis (Kozlowska et al., 2022; Franczak et al., 2022; Koziorowska et al., 2018; Drzewiecka et al., 2021b), which can indirectly affect cell proliferation in uterine tissues. Chronic hormonal imbalances, especially in environments with rich estrogens, are well known to contribute to the development of tumors, which are highly affected by hormonal imbalance, such as uterine fibroids (Ciavattini et al., 2013; Kim et al., 2013). Uterine leiomyoma (LM), also called fibroids, is an example of such tumors developed from clonal expansion of myometrial smooth muscle cells, affected by estrogens and progesterone (Giuliani et al., 2020). Furthermore, epigenetic modifications can result in gene silencing and tumor progression, especially LM development in women, which currently is one of the main indications for hysterectomy (Yang et al., 2016; Mlodawska et al., 2022; Wu et al., 2024; Suo et al., 2022). So far, however, studies investigating the effects of ELF-EMF exposure on these modifications in LMs remain unknown. Therefore, it cannot be excluded that the ELF-EMF-induced hormonal disruptions (Kozlowska et al., 2022; Koziorowska et al., 2018) could potentially create a permissive environment for fibroids growth (Kozlowska et al., 2022; Koziorowska et al., 2018). Furthermore, it has been suggested in several studies that hormonal secretion disruption such as excessive E2 signalling and reduced P4 signalling in the endometrium can lead to various disease and pathological conditions like abnormal endometrial proliferation and differentiation which can develop to endometriosis and adenomyosis (Pru, 2022; Yu et al., 2022; Singh et al., 2022). The ELF-EMF may promote abnormal cell proliferation and differentiation in the various organs and tissues, which could predispose these tissues to the development of benign or malignant growths (Panagopoulos et al., 2021; Nezamtaheri et al., 2022). This is consistent with studies that suggest, the ELF-EMF exposure may promote tumor development and impact female fertility, as observed in animal models where long-term exposure resulted in reduced fertility and altered cell proliferation (Qi et al., 2015). Also, it has been shown that ELF-EMF exposure induces cell proliferation and DNA damage in both normal and tumor cells, highlighting how the ELF-EMF can promote the initial stages of mutation and DNA strand breakage (Lee et al., 2016).

Moreover, immunosuppression is closely linked to tumorigenesis, as the immune system get weakened it reduces the body’s ability to detect and eliminate abnormal or malignant cells, thereby it can increase the risk of developing cancer (Yürekli and Erbaş, 2021; Wang and DuBois, 2015). It has been also reported in several studies that short-term exposure to ELF-EMF (2–24 h per day for up to a week) generally enhances immune responses, whereas prolonged exposure (2–24 h per day for up to 8 years) predominantly leads to immunosuppression (Drzewiecka et al., 2021a; Mahaki et al., 2019), which can potentially lead to tumor development (Evans et al., 2006; Whiteside, 2006).

Although no direct evidence currently links ELF-EMF exposure to tumorigenesis in the uterus, the potential for ELF-EMF to enhance proliferation of myometrial and endometrial cells requires further investigation, particularly in the context of interactions with known carcinogenic factors. Understanding the potential of the ELF-EMF in influencing cell proliferation is crucial for assessing its long-term implications on uterine biology, and further research is needed to evaluate the potential carcinogenic effects of the ELF-EMF in the uterus. As summarized in Table 1, the studies on ELF-EMF exposure across different experimental models provide a comprehensive view of its biological impact on uterine tissues. The table highlights findings from both in vitro and in vivo investigations in pigs and rodents, demonstrating that ELF-EMF exposure can alter transcriptomic profiles, disrupt epigenetic regulation, modulate steroidogenesis, and affect apoptotic and proliferative pathways in the endometrium and myometrium. Collectively, these studies underscore how ELF-EMF-induced molecular and cellular changes may compromise reproductive health and uterine function.

**TABLE | 1:** The key studies discussed, presenting experimental model (*in vitro, in vivo*), species, ELF-EMF parameters, main biological endpoints, and principal findings in the uterus.

Study	Model	Species/Tissue	ELF-EMF parameters	Exposure duration	Main biological endpoints	Principal findings
Aydin et al., 2009 (Toxicol Ind Health)	*In vivo*	Rat/Uterus	50 Hz	Repeated exposure (days–weeks)	Hormones, histopathology	Chronic exposure was associated with hormonal alterations and histopathological changes in uterine tissue
Koziorowska et al., 2018 (Theriogenology)	*In vitro*	Pig/Endometrium and Myometrium	50 Hz, mT range	Hours	Estradiol-17β synthesis	Altered estradiol synthesis and secretion in the uterus
Kozlowska et al., 2021a (Anim Reprod Sci)	*In vitro*	Pig/Endometrium	50 Hz/120 Hz, mT range	Hours	Androgen synthesis and release	Altered androgen production
Drzewiecka et al., 2021a; Drzewiecka et al., 2021b (Int J Mol Sci)	*In vitro*	Pig/Myometrium	50 Hz, mT range	Hours	Transcriptomic profiling	Differential expression of genes related to contractility and metabolism.
Kozlowska et al., 2022 (Reprod Biol)	*In vitro*	Pig/Endometrium	50 Hz/120 Hz, mT range	Hours	Estrogen synthesis and release	ELF-EMF exposure could disrupt estrogen synthesis and secretion
Wydorski et al., 2023 (Reprod Fertil Dev)	*In vitro*	Pig/Endometrium	50 Hz, mT range	Hours	Global DNA methylation	Changes in global DNA methylation levels
Franczak et al., 2023 (Theriogenology)	*In vitro*	Pig/Myometrium	50 Hz, mT range	Hours	DNA methylation, epigenetic regulators	Changes in DNA methylation level and epigenetic regulators expression.
Franczak et al., 2023 (Theriogenology)	*In vitro*	Pig/Endometrium	50 Hz, mT range	Hours	Epigenetic regulation	Coordinated changes in epigenetic marks and transcription
Wydorski et al., 2024a (Int J Mol Sci)	*In vitro*	Pig/Endometrium	50 Hz, mT range	Hours	Apoptosis and oxidative stress-related genes/proteins	Altered apoptosis- and oxidative stress-related markers.

## 8 Conclusions and perspectives

To sum up, the current available evidences indicate that the ELF-EMF exposure induces significant biological effects in the myometrium and the endometrium. These effects are exerted at multiple levels of cellular regulation, which include epigenetic alterations, disrupted steroidogenesis, enhanced oxidative stress, increased apoptosis, and changes in cell proliferation and myometrial contractility. These disruptions can have adverse effects on reproductive functions, potentially affecting fertility, embryo implantation, pregnancy maintenance, and increasing the risk for uterine pathologies like fibroids or endometriosis, which are worldwide concern.

Despite compelling *in vitro* and animal *in vivo* model data, human studies are still limited and sometimes contradictory compared to the *in vitro* and animal studies. Therefore, we cannot draw definitive conclusions about the ELF-EMF effects on human reproductive health. Future well-controlled research should be implicated, especially long-term *in vivo* studies in populations at greater risk, such as pregnant individuals, for example. Furthermore, investigating potential synergistic effects between ELF-EMF exposure and other environmental stressors could provide more realistic insights into reproductive risk which they can make.

While animal studies have linked the ELF-EMF exposure to reduced delivery rates and increased preterm births, due to the ethical aspects, human data are inconclusive about this issue. This variation highlights the species-specific responses and the need for caution when extending these findings to clinical contexts. Moreover, comprehensive mechanistic studies focusing on ion channel regulation, hormonal balance, and epigenetic markers will be essential to clarify the pathways through which ELF-EMF may impact uterine physiology.

Lastly, this review highlights the importance of interdisciplinary researches to assess the full scope of the ELF-EMF’s effect on reproductive health.
